# Early classification of multivariate temporal observations by extraction of interpretable shapelets

**DOI:** 10.1186/1471-2105-13-195

**Published:** 2012-08-08

**Authors:** Mohamed F Ghalwash, Zoran Obradovic

**Affiliations:** 1 Center for Data Analytics and Biomedical Informatics, Temple University, Philadelphia, USA; 2 Mathematics Department, Faculty of Science, Ain Shams University, Cairo, Egypt

## Abstract

**Background:**

Early classification of time series is beneficial for biomedical informatics problems such including, but not limited to, disease change detection. Early classification can be of tremendous help by identifying the onset of a disease before it has time to fully take hold. In addition, extracting patterns from the original time series helps domain experts to gain insights into the classification results. This problem has been studied recently using time series segments called *shapelets*. In this paper, we present a method, which we call *Multivariate Shapelets Detection (MSD)*, that allows for early and patient-specific classification of multivariate time series. The method extracts time series patterns, called *multivariate shapelets*, from all dimensions of the time series that distinctly manifest the target class locally. The time series were classified by searching for the earliest closest patterns.

**Results:**

The proposed early classification method for multivariate time series has been evaluated on eight gene expression datasets from viral infection and drug response studies in humans. In our experiments, the MSD method outperformed the baseline methods, achieving highly accurate classification by using as little as 40%-64% of the time series. The obtained results provide evidence that using conventional classification methods on short time series is not as accurate as using the proposed methods specialized for early classification.

**Conclusion:**

For the early classification task, we proposed a method called Multivariate Shapelets Detection (MSD), which extracts patterns from all dimensions of the time series. We showed that the MSD method can classify the time series early by using as little as 40%-64% of the time series’ length.

## Background

In medical informatics, the patient’s clinical data records, such as heart rate, are collected over time and therefore represent a time series. If the data is collected from two groups of patients (for example, symptomatic and asymptomatic with respect to heart failure), the task of multivariate time series (MTS) classification is to learn temporal patterns to determine whether the patient belongs to the group of symptomatic patients.

Time series have been extensively analyzed in various fields, such as statistics, signal processing, and control theory. The focus of the research in these fields is on gaining a better understanding of the data-generating mechanism, the prediction of future values, or the optimal control of a system. From a statistical viewpoint, time series analysis is comprised of methods for analyzing time series data in order to extract meaningful statistics from the data. As a part of time series analysis, time series forecasting is aimed to use a model, e.g. AutoRegressive Moving Average (ARMA), to predict future values based on previously observed values [1]. The ultimate objective of the signal processing community is the characterization of the time series in such a manner as to allow for transformation of the time series, with a method like Fast Fourier Transformation (FFT), to extract useful information from the time series [2]. Researchers and practitioners in Control Theory strive to calculate solutions for proper corrective action from the controller (inputs) that result in system stability. A set of past inputs and outputs is observed, and new inputs are set in such a way as to try to achieve a desired output [3].

Although all of the aforementioned methods could be helpful in our study, and the experience of researchers and practitioners from other fields are extremely valuable, the focus of our research is to classify a new time series as early as possible by looking at and extracting patterns from past observations rather than predicting future values or analyzing a single time series’ pattern.

In the data mining community, the time series classification problem has been studied in some detail as well. The predictive patterns framework has been introduced to directly mine a compact set of highly predictive patterns [4]. Instead of adopting a two-phase approach by generating all frequent patterns in the first phase and selecting the discriminative patterns in the second phase, this approach integrates pattern mining and feature pruning into the same phase to filter out non-informative and redundant patterns while they are being generated. A temporal rule-based classification method for temporal pattern representation was recently proposed to address the deficiencies of existing methods [5].

A method that extracts all meta-features from a multivariate time series was proposed by Kadous et al. [6]. The types of meta-features are defined by the user, but are extracted automatically and are used to construct propositional attributes (attribute-value features) for another high-level classifier, like a decision tree, that learns a non-linear hypothesis to distinguish among classes.

In the context of classification of unknown time series (time series with an unknown label), models utilize the whole time series with the unknown label to predict it based on the information learned from training data. In an early classification context, the objective is to provide patient-specific classification of unknown time series as early as possible. Therefore, instead of utilizing the whole time series, our MSD method looks into a portion (current stream) of the unknown time series and determines whether it is able to predict the label of the whole time series without looking at the rest of the time series. If MSD is able to predict at the time point which is at the end of the current stream, the label is predicted. Otherwise, MSD requires more data for the unknown time series and looks at a larger segment, and does so until it is able to predict the label of the time series.

For early classification, a new method called *Early Classification on Time Series* (ECTS) has been proposed [7]. The idea behind the method is to explore the stability of the nearest neighbor relationship in the full space and in the subspaces formed by prefixes of the training examples. The disadvantage of ECTS is that it only provides classification results, without extracting and summarizing patterns from training data; thus, users may not be able to gain deep insights from the classification results. This drawback of ECTS has been resolved by extracting local shapelets which distinctly manifest the target class locally, and are effective for early classification [8]. However, the method is applicable only to one-dimensional time series.

In this study, we generalize the definition of local shapelets to a multivariate context and accordingly propose a method for early classification of multivariate time series. The proposed method is called *Multivariate Shapelets Detection* (MSD). A multivariate shapelet consists of multiple segments, where each segment is extracted from exactly one dimension. The test time series is then classified based on the multivariate shapelets that best match the test time series.

In particular, we propose the following extensions to the existing univariate shapelet method: 

• Extending the concept of univariate shapelets to multivariate shapelets, which are multidimensional subsequences with a distance threshold along each dimension.

• Proposing use of information gain-based distance threshold.

• Proposing use of weighted information-gain based utility score of a shapelet. A theorem is provided to show that the weighted information gain incorporates the earliness and assigns high utility score to the shapelet that appears earlier given the same accuracy performance.

The mathematical definition of the problem is presented in the Definitions section. The method for multivariate time series classification is described in the Methods section. Datasets are described in the Dataset and data processing section. In the Results and discussion section, the experimental results are presented. Finally, future work and concluding remarks are discussed in the Conclusion section.

### Definitions

A time series *T* = {*t*_1_, *t*_2_, …, *t*_*L*_} of length *L*, *len*(*T*) = *L*, is defined as a sequence of real values sampled at *L* time stamps. Each time series is associated with a class label *c *∈ *C*where *C* is a finite set of class labels. A dataset *D* is a collection of *M* pairs {(*T*_*i*_, *c*_*i*_) : *i* = 1…*M*} where *T*_*i*_ is the time series number *i* and *c*_*i*_ = *Class*(*T*_*i*_) is its class. Given a time series *T* = {*t*_1_, *t*_2_, …, *t*_*L*_}, a subsequence *s* = {*t*_*i*_, *t*_*i* + 1_, …, *t*_*i* + *l*− 1_}, s⊂T, is a sampling of contiguous positions of *T* of length *l* <*L*. Given two subsequences *s* and *h* where *len*(*s*) = *len*(*h*) = *l*, the Euclidean distance between *s* and *h* is defined as:

(1) $$\text{dist}(s,h)=\sqrt{\overset{l}{\underset{k=1}{\sum }}{\left(s[k]-h[k]\right)}^{2}}$$

**Figure 1:**
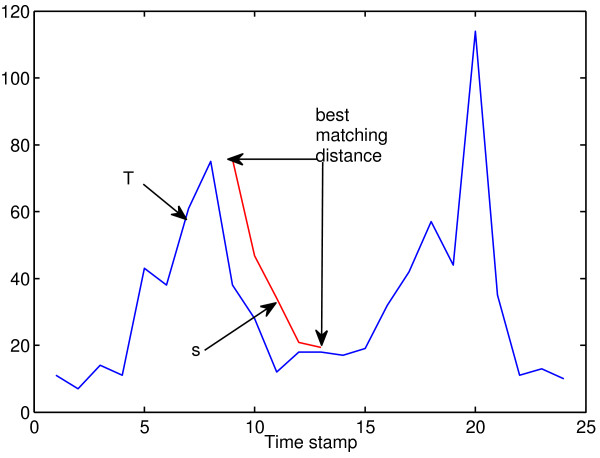
**Illustration of computing the distance between a subsequence*s*and a time series*T*.** To compute the distance between a subsequence *s* of length 5 and a time series *T* of length 24, a window of length 5 is slid over *T* and the distance between *s* and *T* is computed as the minimum distance between *s* and every subsequence of *T* with length 5.

For a given time series *T* of length *L* and a subsequence *s* of length *l*, the distance between *s* and *T* is defined as the minimum distance between *s* and all subsequences of *T* of length *l*. Therefore, we slide a window of length *l* over the time series *T* to extract all subsequences {*h*_1_, *h*_2_, … *h*_*L* − *l* + 1_} of length *l*. As shown in Figure 1, the distance between *s* and *T* is computed as:

(2) $$\text{dist}(s,T)=\underset{\forall i\in \{1,2,\ldots ,L-l+1\}}{\text{min}}\text{dist}(s,h_{i})$$

**Figure 2:**
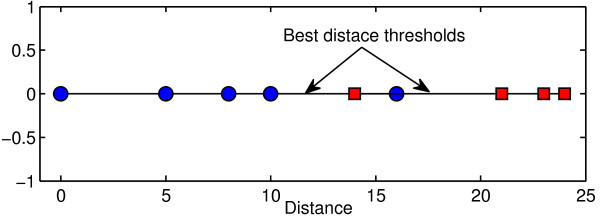
**Illustration of the distance threshold.** The distance threshold is chosen such that it divides the dataset into two separate groups (red and blue groups). It is clear that there is no unique best threshold. Any threshold between 10 and 14 or between 16 and 21 has only either one false negative or one false positive. However, there is no perfect threshold that separates the datasets into two pure groups.

A shapelet is defined as *f* = (*s*,*l*,*δ*,*c*_*f*_) where *s* is a time series subsequence of length *l*. The class label *c*_*f*_ of the shapelet is called the target class. The other classes are called the non-target classes, and are referred to as ${\bar{c}}_{f}$. We call a time series *T*_*i*_ a target time series if the class of the time series is *c*_*f*_. The distance threshold *δ*is computed as follows: 

• The distance *d*_*i*_between *s* and every time series *T*_*i *_ in the dataset is computed using Equation 1. The distance *d*_*i *_ is represented as a point in the order line as shown in Figure 2. If *Class*(*T*_*i*_) = *c*_*f*_, then *d*_*i*_ is represented as blue point. If *Class*(*T*_*i*_) ≠* c*_*f*_, then *d*_*i *_is represented as red square.

• The distance threshold *δ*is computed (as explained in the Methods section) to separate the two groups (blue and red groups).

In another way, the distance threshold *δ* is computed such that the distance between any target time series *T*_*i*_ and *s* is less than the threshold *δ*:

(3) $$\forall (T_{i},c_{f})\in D\Rightarrow \text{dist}(s,T_{i})\leq \delta$$

The distance between a shapelet *f * and time series *T* is defined as *dist*(*f*,*T*) : = *dist*(*s*,*T*).

An *N*-dimensional (multivariate) time series of length *L* is defined as **T** = [*T*^1^,*T*^2^,…,*T*^*N*^] where *T*^*j*^ is the *j*^*th*^ dimension of **T** and *T*^*j*^ [*k*] is the value of the *j*^*th*^ dimension of **T** at time stamp *k*. Hereafter, we use the terms ‘multidimensional’ and ‘multivariate’ interchangeably.

**Figure 3:**
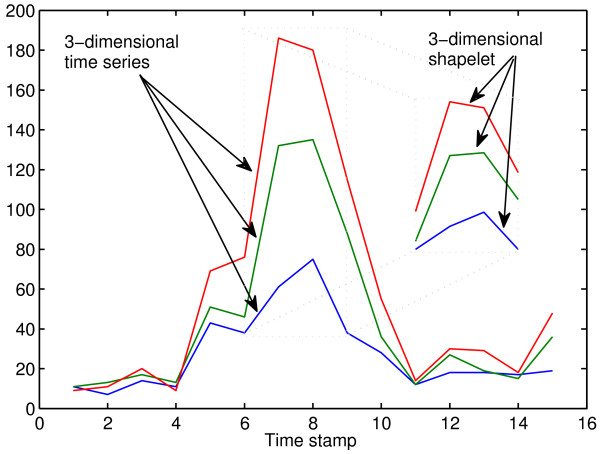
**Illustration of a 3-dimensional shapelet.** This shows an example of a 3-dimensional time series (red, green and blue lines) of length 15. An example of an extracted 3-dimensional shapelet of length 4 is illustrated in the right part of the figure. The shapelet is extracted from the time series from position 6 to position 9.

An *N*-dimensional shapelet (*N*-shapelet) of length *l* is defined as **f** = (**s**,*l*,*Δ*,* c*_*f*_). The vector **s** = [*s*^1^,*s*^2^,…,*s*^*N*^] where *s*^*j*^ is the *j*^*th*^ dimension of the shapelet. Figure 3 shows an example of a 3-dimensional time series of length 15. It shows an example of an extracted 3-dimensional shapelet of length 4. The shapelet is extracted from the time series from position 6 to position 9.

The distance between an *N*-shapelet **f** and *N*-dimensional time series **T** is a vector of *N* Euclidean distances and is defined as:

(4) $$\begin{array}{c} \;\text{dist}(s,T)=\left[\text{dist}\left(s^{1},T^{1}\right),\text{dist}\left(s^{2},T^{2}\right),\ldots ,\text{dist}\left(s^{N},T^{N}\right)\right] \end{array}$$

where *dist*(*s*^*j*^,*T*^*j*^) is defined as in Equation 1. Simply, the distance between two multivariate time series is a vector of distances where each component in the distance vector is the distance between the corresponding dimensions of the two multivariate time series. The distance between a shapelet **f** and a time series **T**is defined as *dist*(**f**,**T**) := *dist*(**s**,**T**).

The distance threshold Δ = [*δ*^1^,*δ*^2^,…,*δ*^*N*^] where *δ*^*j*^ is computed (as explained in the Methods section) so that:

(5) $$\forall (T_{i},c_{f})\in D\Rightarrow \text{dist}(s^{j},T_{i}^{j})\leq \delta^{j}\;\;\forall j=1\ldots N$$

## Methods

In this section we first describe a recently proposed method for early classification of univariate time series [8] together with our suggested modifications. Then, we propose a new method for early classification of multivariate time series.

### Modifications of univariate shapelet for early time series classification

An *Early Distinctive Shapelet Classification (EDSC)* method, which is proposed at [8] and described in Algorithm 1, is aimed to extract a small set of shapelets from univariate time series for early classification.

### Algorithm 1: UnivariateShapeletsDetection

**Input**: A training dataset D of M univariate time series; *minL*; *maxL*

**Output**: A list of univariate shapelets 

1. **for***each time series T* ∈ D **do** {*T* is of length *L*}

2. **for***l* ← *minL***to***maxL***do** {for each shapelet length}

3. **for***k* ← 1 **to***L* – *l* + 1 **do** {for each starting position}

4. ShapeletDist(*k,l,*)

5. ComputeThreshold (*f*_*lk*_,)

6. ComputeUtilityScore (*f*_*lk*_)

7. Add(*f*_*lk*_, *ShapeletList*)

8. PruneShapelets(*ShapeletList*)

9. **return***ShapeletList*

The method iterates over the time series in the dataset *D* (line 1). For each time series *T*, all shapelets of length *l* between *minL* and *maxL* (user parameters) are extracted from *T*. For each shapelet *f*_*lk*_ (lines 2 and 3) the method calls the function *ShapeletDist* (line 4) that computes the distances between *f*_*lk*_ and all time series in *D* using Equation 1. Then, the method computes the distance threshold (line 5) for the candidate shapelet *f*_*lk*_ using Chebyshev’s inequality. Then, it assigns *f*_*lk*_ a utility score (line 6) using a weighted *F*_1_ score measure. In line 8, the method ranks all extracted shapelets using their utility scores and selects a subset of the highest ranked shapelets as the pruned set of shapelets which can exhaustively classify time series.

The functions that compute the distance threshold and utility score are explained in the following sections. We describe how to prune the shapelets and use them for early classification in the Shapelet Pruning and Classification sections, respectively.

#### Distance threshold method

The Chebyshev’s inequality method is proposed for computing the distance threshold [8]. It guarantees that for any distribution, no more than 1/*b*^2^ of the distribution’s values are more than *b* standard deviations away from its mean [9]. The Chebyshev’s inequality is applied to the non-target time series distances to compute the range where the non-target distance has a low probability of appearing. The method refers to a one-sided test, and is not able to find the distance threshold that can discriminate among the classes well. Here we proposed information gain [10] to find a discriminant distance threshold. In Additional file 1: Table S.4 of the supplementary document, we showed that using information gain as a method to compute the distance threshold outperformed the Chebyshev’s inequality method.

Information gain-based distance threshold for univariate shapelets

The basic idea is to find the shapelet’s distance threshold that maximizes the information gain and divides the dataset into two groups, target and non-target time series [10].

**Figure 4:**
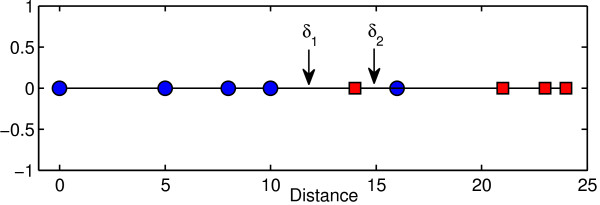
**Candidate distance threshold.** The distance threshold *δ*_1_ splits the dataset into two datasets so that it has 4 true positives, 0 false positive, 4 true negatives, and 1 false negative. The information gain of *δ*_1_ is 0.4090. The distance threshold *δ*_2_ divides the dataset into two datasets so that it has 4 true positives, 1 false positive, 3 true negatives, and 1 false negative. The information gain of *δ*_2_ is 0.1591. Hence, *δ*_1_ has better information gain than *δ*_2_.

First, the entropy of the dataset is computed as

(6) $$\text{Entropy}=-\underset{c\in C}{\sum }\frac{m_{c}}{M}\text{log}\left(\frac{m_{c}}{M}\right)$$

where *m*_*c*_ is the number of time series of class *c* and *M* is the number of all time series. To compute the distance threshold, the method sorts the distances between the shapelet and all time series. Then, it finds the mid point between two consecutive distances as a candidate for the threshold. The dataset is then divided into two datasets *D*_*L*_ and *D*_*R*_ as illustrated in Figure 4. The dataset *D*_*L*_ contains all time series such that the distance between the shapelet and time series is less than or equal to the candidate threshold. The dataset *D*_*R*_ contains the rest of the time series. Then the entropies *E*_*L*_ and *E*_*R*_ of the datasets *D*_*L*_ and *D*_*R*_ are computed, respectively. By comparing the entropy before and after the split, we obtain a measure of information gain which is computed as

(7) $$\text{IG}=\text{Entropy}-\frac{M_{L}}{M}E_{L}-\frac{M_{R}}{M}E_{R}$$

where *M*_*L*_ and *M*_*R*_ are the number of time series in *D*_*L*_ and *D*_*R*_. Therefore, we choose the distance threshold that maximizes the information gain for the shapelet. The algorithm is described in details in Additional file 1: Algorithm S.2.

Figure 4 shows an example of two distance thresholds *δ*_1_ and *δ*_2_. The threshold *δ*_1_ splits the dataset into two datasets so that it has 4 true positives, 0 false positive, 4 true negatives, and 1 false negative. The information gain of *δ*_1_ is 0.4090. The distance threshold *δ*_2_ divides the dataset into two datasets so that it has 4 true positives, 1 false positive, 3 true negatives, and 1 false negative. The information gain of *δ*_2_ is 0.1591. Therefore, the threshold *δ*_1_ is chosen because it has maximum information gain.

#### Utility score method

The set of shapelets extracted from the dataset might be exceedingly large. Therefore, it is important to rank the shapelets in order to select a small subset of the shapelets for classification. For this reason, each shapelet has to be assigned a score that takes into consideration earliness as well as discrimination among classes.

The weighted *F*_1_ score method is proposed to rank shapelets [8]. In our study, we introduce the weighted information gain as a new utility score method. In the supplementary document (Additional file 1: Table S.5) we showed that our proposed method outperformed the weighted *F*_1_ method.

Weighted information gain

The utility score of a shapelet should incorporate the earliness and the distinctiveness properties. First, we define the earliness [8] between a shapelet *f* = (*s*,*l*,*δ*,*c*_*f*_) and a time series *T* as

(8) $$\text{EML}(f,T)=\underset{\forall i\in \{1,2,\ldots ,L-l+1\}}{\text{min}}\text{dist}(s,h_{i})\leq \delta$$

*EML* measures how early the shapelet *f * has classified the time series *T*. The weighted information gain of the shapelet is computed as follows: 

1. Compute the distance between the shapelet *f* = (*s*, *l*, *δ*, *c*_*f*_) and every time series *T*_*i*_in the dataset.

2. Split the dataset *D* into two datasets *D*_*L*_ and *D*_*R*_ such that *D*_*L*_ contains all time series where *dist*(*f*, *T*_*i*_) ≤ *δ* and *D*_*R*_ contains all time series where *dist*(*f*, *T*_*i*_) >*δ*.

3. For each time series *T* in the dataset *D*_*L*_, if *Class*(*T*) = *c*_*f*_, then *T* is weighted by *EML*(*f*,*T*). Otherwise, the time series is weighted by 1.

4. Compute *M*_*L*_ as the weighted count of the number of time series in the dataset *D*_*L*_ and *M*_*R*_ is the size of the dataset *D*_*R*_.

5. Compute the weighted information gain using Equation 4.

The following theorem proves that the weighted information gain incorporates the earliness and assigns high utility score to the shapelet that has better earliness given the same accuracy performance.

 Theorem: If *f*_1_ and *f*_2_ are two shapelets that have the same distance threshold (same splitting point), the same class, and different earliness (*f1* has better earliness than *f2*), then *f1* has better weighted information gain than *f2*. Proof: Suppose that the number of target time series in *D*_*L*_ is *N*_*T*_ and the number of non-target time series in *D*_*L*_ is *N*_*NT*_. Without loss of generality, since *f1* has better earliness than *f2*, suppose that for every target time series *T* in *D*_*L*_, *EML*(*f*_1_,*T*) = *P*_1_ and *EML*(*f*_1_,*T*) = *P*_2_ such that *P*1 <*P*2. The weighted count *M*_*L*1_ and *M*_*L*2_ of the time series in *D*_*L*_ for *f*_1_ and *f*_2_ is *P*_1_*N*_*T*_ + *N*_*NT*_ and *P*_2_*N*_*T*_ + *N*_*NT*_, respectively. Since *P*1 <*P*2, then *M*_*L*1_ <*M*_*L*2_. Hence the weighted information gain of *f*_1_ is greater than the weighted information gain of *f*_2_.

Therefore, the weighted information gain gives high scores to the shapelets that come early in the time series.

#### Shapelet pruning

To select a subset of the shapelets for classification, the shapelets are sorted in descending order using their utility scores. In this manuscript, two methods have been used to select a subset of the shapelets.

The first method iterates over the shapelets starting from the highest ranked shapelet. We select the shapelet and remove all training examples that are covered by that shapelet. The shapelet *f * covers a training time series *T* if *dist*(*f*,*T*) ≤ *δ* and *Class*(*T*) = *c*_*f*_. We use the next highest ranked shapelet to see if it covers any of the remaining training time series. If it covers some of them, then we select the shapelet and remove all time series that are covered. Otherwise, we discard it and proceed to the next one. This process continues until all training time series are covered.

The second method simply involves keeping the top *x* shapelets from each class where *x* is a user-defined parameter. In our experiments, we used the top 5, 10, 15 and 20 shapelets from each class.

#### Classification

If the length of the shortest shapelets extracted by Algorithm 1 is *l*, then we can not classify any time series before observing *l* time points. Hence, the classification method (Additional file 1: Algorithm S.1) initially reads *l* time stamps from the test time series. It then gets the highest-ranked shapelet. If the shapelet covers the current stream of the test time series then the time series is classified as the class of the shapelet and the prediction is done. Otherwise, it gets the next shapelet from the ranked list and repeats the process. If none of the shapelets cover the current stream of the test time series the method reads one more time stamp and continues classifying the time series. Therefore, the test time series could be classified after reading number of time points greater than the shapelet’s length. If the method reaches the end of the time series and none of the shapelets covers it, the method marks the time series as a not-classified example. In the results section, we report the relative accuracy as well as the percentage of the covered test time series.

### Multivariate shapelets detection for ECMTS

In a dataset of *N*-dimensional time series, the method extracts all *N*-dimensional shapelets **f** = (**s**,*l*,*Δ*,*c*_*f*_). The method assumes that all subsequences *s*^*j*^ are extracted from the same starting position. Hence, we slide a window of length *l* over the time series. At each time stamp *p*, a subsequence *s*^*j*^ of length *l* starting from time point *p* is extracted from the *j*^*th*^ dimension to construct **s** = [*s*^1^,*s*^2^,…,*s*^*N*^]. An example of a 3-dimensional shapelet is shown in Figure 3.

We follow the same procedures as in the univariate case. Namely, for each *N*-shapelet **f**, we compute the minimum distance between **f** and every time series **T** in the dataset. The distance between **f** and **T** is a vector of distances (*N*-dimensional distance) and is computed as in Equation 2. To compute the distance threshold of a shapelet, we need to provide a way to compare two multi-dimensional distances. Therefore, two multidimensional distances $d_{1}=[d_{1}^{1},d_{1}^{2},\ldots ,d_{1}^{N}]$ and $d_{2}=[d_{2}^{1},d_{2}^{2},\ldots ,d_{2}^{N}]$ are defined to be ordered according to the following criterion:

(9) $$d_{1}<d_{2}\Leftrightarrow d_{1}^{j}<d_{2}^{j}\;\;\forall j=1\ldots N$$

Equation 5 requires all *N* dimensions of **d**_1_ to be less than all corresponding *N* dimensions of **d**_2_. Therefore, we would require all *N* dimensions to be less than the shapelet’s threshold. This way, the method would try to find a pattern very similar to the shapelet at hand, which could lead to overfitting. In order to prevent overfitting, Equation 5 is relaxed and redefined to be partially ordered according to the following criteria:

(10) $$d_{1}{<}_{\text{Perc}}d_{2}\Leftrightarrow d_{1}^{q_{j}}<d_{2}^{q_{j}}\;\;\forall j=1\ldots \text{Perc}\times N$$

where *Perc* ∈ ]0,1].

The algorithm for extracting the multivariate shapelets from a dataset is similar to Algorithm 1. The algorithm iterates over each time series and extracts all multivariate shapelets. For each candidate multivariate shapelet, it computes the distances with every time series. Note that each distance is a vector of length *N*. Hence, the distances between a multivariate shapelet and all time series is a matrix with dimensions *N* × *M* where M is the number of time series. Then, the method computes the distance threshold and utility score for each candidate multivariate shapelet as explained in the following section. Finally, it prunes the shapelets using the same procedure as mentioned in the univariate case.

#### Distance threshold method

Multivariate information gain-based distance threshold for multivariate shapelets

The multivariate information gain (Additional file 1: Algorithm S.3) is computed in a similar way to the one that computes the information gain in the univariate case. It takes as input an *N*-shapelet **f**; a matrix *Dist*, that stores the multivariate distances between the shapelet and all *M* time series in the dataset; and *Perc*, which determines the percentage of dimensions used to compute Equation 6. It sorts the matrix *Dist*, and then the multivariate candidate threshold is computed as the mid-point between two successive distances (columns in the matrix *Dist*). Using the candidate threshold, the information gain is computed. Finally, the algorithm returns the multivariate threshold *Δ* = [*δ*^1^,*δ*^2^,…,*δ*^*N*^] that has maximal information gain.

#### Utility score method

The steps to adapt the utility scores defined on univariate time series are similar to the steps we have followed to adapt the distance threshold method.

After computing the score for each shapelet, the method sorts them in descending order according to their utility scores and then selects a subset of shapelets as explained in the Shapelet Pruning section. The classification process is similar to the process described in the Classification section, taking Equation 6 into consideration when computing the distance between the shapelet and the current stream of the query time series.

## Dataset and data processing

### Viral challenge datasets

We used two datasets for blood gene expression from human viral studies with influenza A (H3N2) and live rhinovirus (HRV) to distinguish individuals with symptomatic acute respiratory infections from uninfected individuals [11].

H3N2 dataset: A healthy volunteer intranasal challenge with H3N2 was performed in 17 subjects. Of those subjects, 9 became symptomatic and 8 remained asymptomatic. Blood samples were taken from each subject at 16 time points. Some subjects have missed certain measurements at time points 1,5,6 and/or 7. Hence, the gene expression values were measured on average 14-16 times for each subject. 30 genes were identified, in ranked order, as contributing to respiratory infection [11]. We used 23 unique genes from that list that were found in the available dataset.

HRV dataset: A healthy volunteer intranasal challenge with HRV was performed in 20 subjects. Of those subjects, 10 became symptomatic and 10 remained asymptomatic. Blood samples were taken from each subject at 14 time points. We ignored time stamps 8-11 because the majority of the subjects missed the measurements at those time points. Thus, the gene expression values were measured on average 6-10 times for each subject. 30 genes were identified, in ranked order, as contributing to respiratory infection [11]. We used 26 unique genes from that list that were found in the available dataset.

### Drug response dataset

Another clinical dataset was generated for studying the changes in cellular functions in multiple sclerosis (MS) patients in response to drug therapy with IFN *β*[12]. The dataset contains time series gene expression for 52 patients. The patients were classified as good responders (33 patients) or bad responders (19 patients) to the drug. The blood samples were taken every 3 months in the first year and every 6 months in the second year. Some patients missed certain measurements, especially at the 7^*th*^ time point. Thus, the gene expression values were measured on average 5-7 times for each subject. The list of the genes used in our experiments is provided (Additional file 1: Table S.1).

Identification of triplets of genes for a Bayes classifier of time series expression data of multiple sclerosis patients’ response to the drug has been performed [12]. Previous research identified 12 genes in terms of triplets. Hence, we generated four datasets: Baranzini3A and Baranzini3B, consisting of one triplet of the best two triplets of genes, respectively; Baranzini6 has the top two triplets; and Baranzinin12 has all 12 genes identified by all triplets.

A discriminative hidden Markov model has been developed and applied to the MS dataset to reveal the genes that are associated with the good or bad responders to the therapy [13]. A total of 9 genes were found that are associated with the therapy. Hence, we constructed a dataset, called Lin9, consisting of those 9 genes.

A mixture of hidden Markov models has been developed to identify the genes that are associated with the patient response to the treatment [14]. A total of 17 relevant genes were found. Therefore, we constructed a dataset called Costa17 that contains data for these 17 genes.

### Environment setup and evaluation measure

In all experiments we set *minL* = 3 and *maxL* to be 60% of the time series’ length. Since the number of subjects was small, bootstrapping was used for estimating the generalization error [15,16]. We sample with replacement a subset (75%) from the original dataset. We train our model on the sample data and then test it on the subjects that are not used in the training data. This process is repeated 1000 times and the final reported statistics (like relative accuracy) is the median of the statistics over all bootstrap samples. We report the median instead of the average since the distribution of the statistics is skewed and not symmetric.

In the results, we report the median of the accuracy, the coverage (the percentage of the time series that are covered by the method), and the earliness (the fraction of the time series length used for classification). Note that the earliness varied from test example to another. In other words, each test example could be classified at different time point, so that our method is patient-specific and there is no fixed length of the time series used for classification.

Because there is an imbalance in the drug response dataset, the accuracy (*Acc*) is calculated as the average between sensitivity and specificity:

(11) $$\begin{array}{lll} \text{Sensitivity} & =\frac{\text{tp}}{\text{tp}+\text{fn}},\;\;\;\text{Specificity}=\frac{\text{tn}}{\text{tn}+\text{fp}},\; & \;\\ \text{Acc} & =\frac{\text{Sensitivity}+\text{Specificity}}{2}\; & \; \end{array}$$

where *tp* is the number of true positives, *tn* is the number of true negatives, *fp* is the number of false positives, and *fn* is the number of false negatives.

Since the objective of the paper is to provide a method for early classification, we propose an evaluation measure that incorporates both the earliness (*Ear*) and the accuracy (*Acc*). We use *F*_*β*_-measure as the weighted average between *Acc* and *Ear*. *F*_*β*_-measure is defined as:

(12) $$F_{\beta }=\left(1+\beta^{2}\right)\frac{\text{Acc. }(1-\text{Ear})}{\beta^{2}(1-\text{Ear})+\text{Acc}}$$

where smaller values of *β* put more weight on the earliness and larger values of *β* put more weight on the accuracy. Note that we use (1−*Ear*) because we want to penalize larger values of *Ear*. In our experiments, we used the balanced *F*_1_-score, which gives both the accuracy and the earliness the same weight. *F*_1_-score reaches its best value at 1 and worst score at 0.

## Results and discussion

### Evaluation of MSD method

**Figure 5:**
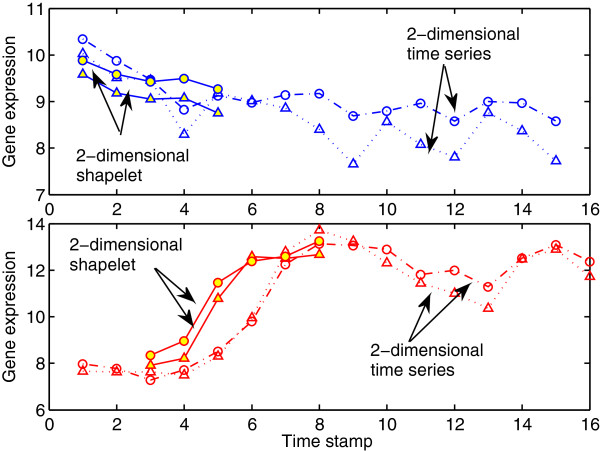
**Illustration of the effectiveness of the MSD method on a case from H3N2 dataset.** The effectiveness of the MSD method is illustrated on a single patient from H3N2. In the top panel, a 2-dimensional H3N2 asymptomatic test subject (genes RSAD2 and IFI44L observed at 15 time steps) has been correctly classified by MSD method at the 5^*th*^ time point. In the bottom panel a 2-dimensional H3N2 symptomatic test subject (genes RSAD2 and IFI44L observed at 16 time steps) has been correctly classified by MSD method at the earliest possible time stamp number 8. Red lines represent time series of the symptomatic subject. Blue lines represent time series of the asymptomatic subject. Shapelets are represents by solid markers.

First, we show the effectiveness of the MSD method on a single patient from the H3N2 dataset. In Figure 5, the top panel shows genes RSAD2 and IFI44L observed at 15 time steps for an asymptomatic test subject from H3N2 data that is correctly and early classified by MSD at the 5^*th*^ time point. The MSD method used a shapelet of length 5 to classify the test subject. In the bottom panel, MSD used a shapelet of length 6 that was extracted from the time series of a symptomatic subject, so it correctly classified the symptomatic test subject at the 8^*th*^ time point (it used only 50% of the time series’ length to classify the test subject).

Next, the MSD method was evaluated on the viral and drug response datasets using all genes defined by the dataset. In Table 1, we report the median of the coverage, the relative accuracy, and the earliness. The list of the parameters that have been used for each method is provided in Additional file 1: Table S.2.

**Table 1:** Evaluation of the MSD method on the viral infection and drug response datasets using all genes

**Dataset**	**Number of genes**	**Accuracy**	**Relative accuracy**	**Coverage**	**Earliness**	***F***_**1**_
H3N2	23	77.78	85.71	100	62.50	0.5060
HRV	26	70.00	71.43	100	40.00	0.6462
Baranzini3A	3	70.00	73.91	95.83	46.26	0.6080
Baranzini3B	3	66.67	68.00	100	44.81	0.6039
Baranzini6	6	70.83	70.83	100	42.86	0.6325
Baranzini12	12	66.67	66.67	100	42.86	0.6154
Lin9	9	67.86	69.57	100	44.00	0.6136
Costa17	17	68.00	69.23	100	45.24	0.6067

The performance of the MSD method on 8 datasets is shown in the table. The MSD method achieved good accuracy on most of the datasets using a small fraction of the time series. The distribution of the statistics were skewed and not symmetric, so we report the median of the statistic.

From Table 1, it is clear that the MSD method achieved high accuracy using a small fraction of the time series. For example, MSD on the H3N2 dataset covered approximately 100% of the dataset, and out of the covered time series it achieved 85.71% accuracy using 62% of the time series’ length. On another benchmark dataset called Lin9, the method developed in [13] achieved 85% accuracy using the full time series (*F*_1_ ≈ 0.01) while our MSD method achieved approximately 68% accuracy using less than half of the time series’ length on average (*F*_1_ ≈ 0.51).

**Figure 6:**
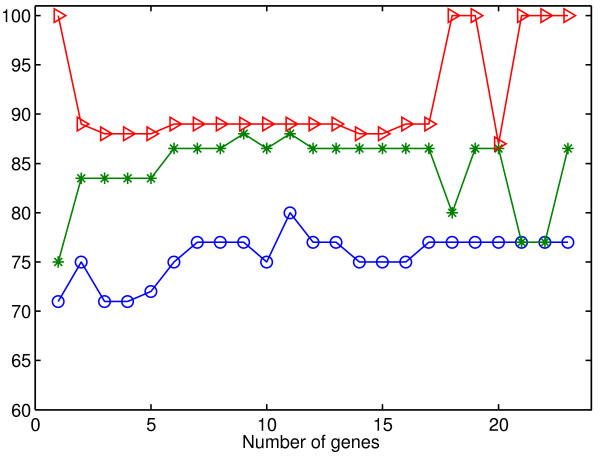
**Performance of MSD method on the H3N2 dataset using different numbers of top genes.** This figure illustrates the performance of the MSD method on the H3N2 dataset using different numbers of top genes from the provided ranked list [11]. Red, green, and blue lines represent coverage, relative accuracy, and accuracy, respectively.

For the viral infection dataset, a list of 23 genes associated with the viral infection sorted by their relevance to the infection diagnosis is provided in a recently published study [11]. Starting from this list, we searched for a subset of genes that could be used to achieve more accurate results. We ran MSD using different numbers of top genes provided by the ranked list. The coverage, the relative accuracy, and the accuracy of MSD on H3N2 are shown in Figure 6. It is clear that the method becomes more accurate when using 11 genes instead of using 23 genes.

For the drug response dataset, no ranked list of genes is provided in previous publications. In 4 out of the 6 drug response datasets the number of the genes is small, therefore, on these datasets, we ran our MSD method on all combinations of genes. The number of genes used for each dataset to achieve the highest accuracy is provided in Table 2. The accuracy of the MSD method on those datasets is improved by using less number of genes. For example, the accuracy of MSD on the Lin9 dataset using only two genes is significantly improved (*F*_1_-score increased from 0.61 to 0.67).

**Table 2:** Evaluation of the MSD method on the drug response datasets using a subset of genes that gives the highest accuracy

**Dataset**	**genes**	**Accuracy**	**Relative accuracy**	**Coverage**	**Earliness**	***F***_**1**_
H3N2	Top 11 genes	80.00	87.50	88.89	64.29	0.4938
HRV	RSAD2	71.43	75.00	100	38.89	0.6587
Baranzini3A	Caspase 10	75.00	76.00	100	45.45	0.6316
Baranzini3B	Caspase 2 , Caspase 3	75.00	76.19	100	44.05	0.6409
Baranzini6	Caspase 10 , IL-4Ra	75.00	76.00	100	43.45	0.6448
Lin9	Caspase 2, Caspase 3, Jak2	81.82	82.61	100	43.43	0.6689

The MSD method has been evaluated on all combinations of the genes on 4 datasets. The accuracy of the classifier is improved than using all genes. For example, the performance of MSD method on the Lin9 dataset is improved significantly from 68% to 82% when using only 3 genes instead of 9 genes.

Since our method achieved high accuracy using a small number of genes (in some cases only one gene), we ran the univariate method [11] (using the Chebyshev’s inequality as distance threshold method and the weighted recall as utility score method) on each gene in the dataset and report the best accuracy achieved. As shown in Table 3, our methods significantly outperformed the univariate method on all datasets except the H3N2 dataset, where they have similar accuracy but the univariate method is much earlier. The reason of achieving less accurate results using MSD method as compared to the univariate method may be due to the non-robustness of the MSD method to noisy variables so that MSD does not extract meaningful features from the multivariate data in an automated fashion. Therefore, Equation 6 is affected by the noise in the variables which may lead to poor discrimination among the classes. In future work, we will investigate more resilient multivariate shapelet detection techniques that effectively utilize a subset of the variables providing maximum discrimination power as compared to using all the variables.

**Table 3:** Evaluation of the univariate method on all datasets

**Dataset**	**gene**	**Accuracy**	**Relative accuracy**	**Coverage**	**Earliness**	***F***_**1**_
H3N2	LOC26010	77.78	85.71	100	38.34	0.6879
HRV	RSAD2	42.86	80.00	55.56	52.50	0.4506
Baranzini3A	Caspase 10	12.00	100.00	12.25	42.86	0.1983
Baranzini3B	Caspase 3	26.09	80.00	31.38	40.26	0.3632
Baranzini6	Caspase 10	12.00	100.00	12.25	42.86	0.1983
Baranzini12	Caspase 3	26.09	80.00	31.38	40.26	0.3632
Lin9	Caspase 3	26.09	80.00	31.38	40.26	0.3632
Costa17	Caspase 3	26.09	80.00	31.38	40.26	0.3632

The univariate method (using the Chebyshev’s inequality as distance threshold method and the weighted recall as utility score method) has been evaluated on each gene on all datasets. The best accuracy is reported.

### Baseline classifier for early classification

We compared the MSD method with a random classifier to evaluate MSD by comparison. The results of the random classifier are shown in Table 4. It is clear that the MSD method is much accurate than the random classifier.

**Table 4:** Evaluation of the random classifier on all datasets

**Dataset**	**Accuracy**
H3N2	55.2833
HRV	52.1869
Baranzini3A	49.7893
Baranzini3B	49.6808
Baranzini6	50.8227
Baranzini12	53.9255
Lin9	50.7689
Costa17	51.5093

In addition, we compared MSD to the baseline classical classifier, which uses shorter time series. Recent research strongly suggested that the 1-nearest neighbor (1NN) method with Dynamic Time Warping (DTW) is exceptionally difficult to beat [17]. Therefore, we compared MSD to the 1NN classifier using DTW. We compared (data is not shown) 1NN using Euclidean distance to 1NN using DTW and we found that 1NN with DTW is more accurate than 1NN with Euclidean distance.

**Figure 7:**
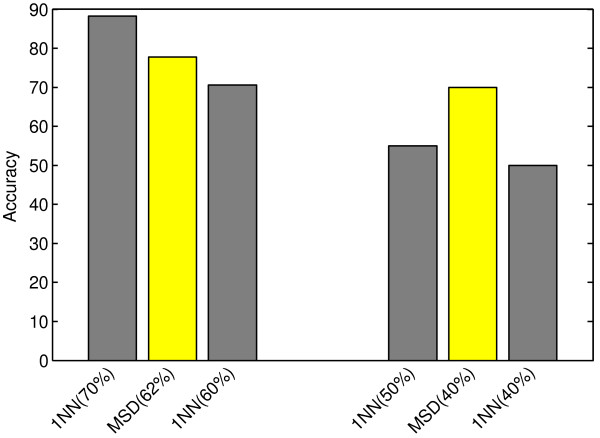
**Comparison of the MSD method to the baseline classifier.** The performance of 1NN with DTW using different time series length and MSD on the viral infection datasets. The left (right) group shows accuracy of the classifiers on H3N2 (HRV) dataset, respectively. The x-axis within a group is ordered by the fraction of the time series, shown in parenthesis. The results provide evidence that the MSD method is more accurate than 1NN.

We constructed 2 datasets out of H3N2, which we call 1NN(70) and 1NN(60). We also constructed 2 datasets out of the HRV dataset, which we call 1NN(50) and 1NN(40). The 1NN(*k*) dataset was constructed from the prefixes of the original dataset such that all its time series are of fraction *k* of the original time series. For each dataset, 1NN was applied using all genes. The results are shown in Figure 7.

On the HRV dataset (right group), the accuracy of 1NN using 50% of the time series’ length (gray bar) is worse than our early classification method MSD (yellow bar), and MSD used a smaller fraction of time series on average. For instance, 1NN achieved 55% accuracy on 1NN(50) dataset (*F*_1_ ≈ 0.46) while MSD was more accurate using on average 40% of time series’ length (*F*_1_ ≈ 0.64). The results were consistent with the H3N2 dataset.

Therefore, for the early classification task, using conventional classification methods on shorter time series is not as accurate as using methods specialized for early classification, such as our proposed method.

### Run-time analysis

In Table 5, we show the run time of the MSD method on viral infection and drug response datasets. All experiments were conducted on a PC Intel Core i7 2.8 GHz with 8GB RAM. It is evident that the run time grows exponentially with the number of examples and the time series length.

**Table 5:** Run-time analysis of MSD on the viral infection and drug response datasets

**Dataset**	**Number**	**Number**	**TS length**	**Time**
	**of genes**	**of examples**		**in seconds**
H3N2	23	17	16	295.1
HRV	26	20	10	77.7
Baranzini3A	3	52	7	49.3
Baranzini3B	3	52	7	36.1
Baranzini6	6	52	7	41.1
Baranzini12	12	52	7	64.3
Lin9	9	52	7	48.8
Costa17	17	52	7	131.9

The run time of the MSD method is reported for all datasets. The number of genes, number of examples, the time series length, and the run time in seconds are reported in the table.

## Conclusion

For the early classification task, we proposed a method called Multivariate Shapelets Detection (MSD). It extracts patterns from all dimensions of the time series. In addition, we proposed using of information gain-based distance threshold and weighted information-gain based utility score of a shapelet. The weighted information gain incorporates the earliness and assigns high utility score to the shapelet that appears earlier. In order to adhere to the limitations of clinical settings (in which only a small pre-specified number of genes is provided in shorter time series), datasets comprised of fairly short time series were used in reported experiments. However, our method is applicable to any domain. We showed that MSD can classify the time series early by using as little as 40%-64% of the time series’ length. We compared MSD to a baseline classifier and showed that using the method proposed for early classification is more accurate than using conventional methods.

The run time of the MSD method grows exponentially with the number of examples and the length of the time series which limits the applicability of the proposed approach to datasets with smaller number of data instances and/or temporal observations. In practice, this is not a limitation for early classification in many health informatics applications (e.g. sepsis) since decisions typically have to be made very early by learning from a small number of patients. However, in future work, we will speed up the run time of the method by incorporating parallelism in the algorithm.

We are working to improve MSD by allowing the components of the multivariate time series shapelet to have different starting positions. Since the number of candidate shapelets grows exponentially, the concept of closed shapelets, and maximal closed shapelets can be introduced to pruning redundant shapelets that are supersets of smaller shapelets. Another extension to our work is to let the horizon between the time stamps in the subjects vary.

## Competing interests

Both authors declare that they have no competing interests.

## Author’s contributions

MG designed the algorithms, implemented software, carried out the analysis, and drafted the manuscript. ZO inspired the overall work, provided advice, and revised the final manuscript. Both authors read and approved the final manuscript.

## Supplementary Material

Additional file 1 **Supplementary document.** The supplementary document (ECMTS-Supp.pdf) contains additional analysis of the obtained results. These details are omitted for lack of space but are consistent with the findings reported here. Click here for file

## References

[B1] Box GEP Jenkins GM Reinsel GC Time Sereis Analysis: Forecasting and Control 2008 Wiley, Chichester

[B2] Bracewell RN The Fourier Transform and Its Applications 1999 3edition. McGraw-Hill Science/Engineering/Math

[B3] Goodwin GC Ramadge PJ Caines PE Discrete time multivariable adaptive control18th IEEE Conference on Decision and Control including the Symposium on Adaptive Processes 1979 335 340

[B4] Batal I Hauskrecht M Constructing Classication Features Using Minimal Predictive Patterns ACM Conference on Information and Knowledge Management 2010

[B5] Dua S Saini S Singh H Temporal Pattern Mining for Multivariate Time Series Classification J Med Imaging and Health Inf 2011 1 2 164 169 10.1166/jmihi.2011.1019

[B6] Kadous MW Sammut C Classification of Multivariate Time Series and Structured Data Using Constructive Induction Machine Learning 2005 58 179 216 10.1007/s10994-005-5826-5

[B7] Xing Z Pei J Yu PS Early Prediction on Time Series: A Nearest Neighbor Approach Proceedings 21st International Joint Conference on Artifical Intelligence 2009 1297 1302

[B8] Xing Z Pei J Yu PS Wang K Extracting Interpretable Features for Early Classification on Time Series Proceedings of 11th SIAM International Conference on Data Mining 2011 439 451

[B9] Allen AO Probability, Statistics, and Queuing Theory with Computer Science Applications 1990 Academic Press

[B10] Mueen A Keogh E Young N Logical-Shapelets: An Expressive Primitive for Time Series Classification Proceedings of ACM SIGKDD International Conference on Knowledge Discovery and Data Mining 2011 1154 1162

[B11] Zaas AK Chen M Varkey J Veldman T III AOH Lucas J Huang Y Turner R Gilbert A Lambkin-Williams R Øien NC Nicholson B Kingsmore S Carin L Woods CW Ginsburg GS Gene Expression Signatures Diagnose Influenza and Other Symptomatic Respiratory Viral Infections in Humans Cell Host and Microbe 2009 6 3 207 217 10.1016/j.chom.2009.07.006 19664979 PMC2852511

[B12] Baranzini SE Mousavi P Rio J Caillier SJ Stillman A Villoslada P Wyatt MM Comabella M Greller LD Somogyi R Montalban X Oksenberg JR Transcription-Based Prediction of Response to IFNSS Using Supervised Computational Methods PLoS Biol 2005 3 1 166 176 10.1371/journal.pbio.0030002 PMC539058 15630474

[B13] Lin T Kaminski N Bar-Joseph Z Alignment and classification of time series gene expression in clinical studies Bioinformatics 2008 24 13 i147 i155 10.1093/bioinformatics/btn152 18586707 PMC2718630

[B14] Costa IG Schönhuth A Hafemeister C Schliep A Constrained mixture estimation for analysis and robust classification of clinical time series Bioinformatics 2009 25 12 i6 i14 10.1093/bioinformatics/btp222 19478017 PMC2687976

[B15] Lendasse A Wertz V Verleysen M Model Selection with Cross-Validations and Bootstraps - Application to Time Series Prediction with RBFN Models Artificial Neural Networks and Neural Information Processing ICANN/ICONIP 2003 2003 Springer-Verlag 573 580

[B16] Jain AK Dubes RC Chen CC Bootstrap Techniques for Error Estimation IEEE Trans Pattern Anal Machine Intelligence 1987 PAMI-9 5 628 633 10.1109/tpami.1987.4767957 21869421

[B17] Ding H Trajcevski G Scheuermann P Wang X Keogh E Querying and mining of time series data experimental comparison of representations and distance measures Proc VLDB Endowment 2008 1 2 1542 1552

