# Supplementary material for: Early classification of multivariate temporal observations by extraction of interpretable shapelets
Source: BMC Bioinformatics. 2012 Aug 8;13:195. doi: 10.1186/1471-2105-13-195 (PMC3475011; doi:10.1186/1471-2105-13-195)
Supplement: Additional file 1 — Supplementary document. The supplementary document (ECMTS-Supp.pdf) contains additional analysis of the obtained results. These details are omitted for lack of space but are consistent with the findings reported here. [file 1471-2105-13-195-S1.pdf]

# Supplementary Materials to Early Classification of Multivariate Temporal Observations by Extraction of Interpretable Shapelets

Mohamed F. Ghalwash[1,2,3] and Zoran Obradovic*[1,2]

[1]Center for Data Analytics and Biomedical Informatics, Temple University, USA
[2]Computer and Information Sciences Department, Temple University, USA
[3]Mathematics Department, Faculty of Science, Ain Shams University, Cairo, Egypt

Email: Mohamed F. Ghalwash - mohamed.ghalwash@temple.edu; Zoran Obradovic*- zoran.obradovic@temple.edu;

*Corresponding author

## 1 Univariate shapelet classification

The method for the classification phase is described in Algorithm S.1. It iterates over each test time series. If the length of the shortest shapelets detected from Algorithm 1 is $l$, then we can not classify any time series before observing $l$ time points. Hence, the method initially reads $l$ time stamps from the test time series (line 3). Then, it gets the highest ranked shapelet (line 7). If the shapelet covers the current stream of the test time series (line 10), then the time series is classified as the class of the shapelet and the prediction is done (line 11). Otherwise, it gets the next shapelet from the ranked list (line 7) and repeats the same process again. If none of the shapelets covers the current stream of the test time series (line 8), the method reads one more time stamp from the test time series (line 14) and continues classifying the time series. If the entire test time series has been read and none of the shapelets cover the test example, it marks the time series as an unclassified example (line 19).

## 2 Information gain-based distance threshold for univariate shapelets

Algorithm S.2, based on information gain, computes the shapelet's distance threshold. The idea is that the shapelet's distance threshold is computed such that the shapelet divides the dataset into two groups; target and non-target time series. Therefore, the shapelet's distance threshold is chosen such that the shapelet has maximum information gain. The method to compute the information gain is described in Algorithm S.2.

**Algorithm S.1:** UnivariateShapeletsClassification

```
Input: A test dataset D of M univariate time series; SSS: sorted set of shapelets
Output: FinalPred: List of M predictions
for Ex ← 1 to M do {Loop for each test example}
    EndStream = false
    Read ShortestShapeletLength time stamps from the time series Ex
    repeat
        Classified = false
        while Not Classified do
            f = GetTopShapelet (SSS) {f = (s, l, δ, c_f)}
            if f is empty then {no available shapelet}
                break
            if dist(f, CurrentStream) ≤ δ then {CurrentStream is the current stream of Ex }
                FinalPred(Ex) = c_f
                Classified = true
        if Not Classified AND Ex has more stream then
            Read one more time stamp
        else if Not Classified then
            EndStream = true
    until Classified OR EndStream
    if Not Classified then
        FinalPred (Ex)=∞ {non classified}
return FinalPred
```

First, the entropy of the dataset is computed as:

$$Entropy = -\sum_{c \in C} \frac{m_c}{M} \log(\frac{m_c}{M}) \tag{1}$$

where $m_c$ is the number of time series of class $c$ and $M$ is the number of all time series. To compute the distance threshold, the method takes two parameters, the shapelet and the distances *RowDist* between the shapelet and all time series in the dataset $D$. It sorts the distances (line 2) and finds the mid-point between two consecutive distances as a candidate for the threshold (line 4). The dataset is then divided into two parts left and right to the threshold. The left part (lines 6-10) has all time series such that the distance between the shapelet and any time series is less than or equal to the threshold. The right part (lines 11-15) has the remainder of the time series. Then, the entropy of the left and right parts are computed (lines 16 and 17, respectively). By comparing the entropy before and after the split, we obtain a measure of information gain (line 18) as

$$IG = Entropy - \frac{M_L}{M} E_L - \frac{M_R}{M} E_R \tag{2}$$

where $M_L$ and $M_R$ are the number of time series in $D_L$ and $D_R$, respectively. We choose the distance threshold that maximizes the information gain for the shapelet (line 21).

**Algorithm S.2:** ComputeThreshold - Information Gain

**Input**: A shapelet $f$, RowDist
**Output**: A distance threshold $\delta$

1 Initialize (tp,fp,tn,fn, maxIG)
2 SortedDist = Sort(RowDist)
3 **for** $j \leftarrow 1$ **to** $M-1$ **do**
4     CandThr = (SortedDist (j) + SortedDist (j+1)) / 2
5     **for** $i \leftarrow 1$ **to** $M$ **do**
6         **if** RowDist *(i)*$\leq$ CandThr **then** {distance between $f$ and $T_i$}
7             **if** $Class(T_i){=}{=}Class(f)$ **then**
8                 tp =tp +1{True Positive}
9             **else**
10                 fp =fp +1{False Positive}
11         **else**
12             **if** $Class(T_i){=}{=}Class(f)$ **then**
13                 tn =tn +1{True Negative}
14             **else**
15                 fn =fn +1{False Negative}
16     $E_L = -\frac{\mathsf{tp}}{\mathsf{tp+fp}}\log(\frac{\mathsf{tp}}{\mathsf{tp+fp}}) - \frac{\mathsf{tp}}{\mathsf{tp+fp}}\log(\frac{\mathsf{tp}}{\mathsf{tp+fp}})$
17     $E_R = -\frac{\mathsf{tn}}{\mathsf{tn+fn}}\log(\frac{\mathsf{tn}}{\mathsf{tn+fn}}) - \frac{\mathsf{tn}}{\mathsf{tn+fn}}\log(\frac{\mathsf{tn}}{\mathsf{tn+fn}})$
18     InforGain = Entropy- $\frac{tp+fp}{N}$ $E_L$- $\frac{tn+fn}{N}$ $E_R$
19     **if** InforGain >maxIG **then**
20         maxIG = InforGain
21         $\delta$=CandThr

## 3 Multivariate information gain

The multivariate information gain is computed in a similar way as in Algorithm S.2. The modifications in Algorithm S.2 are in lines 2, 4 and 6. The modifications have been emphasized in Algorithm S.3. The algorithm takes as its input an $N$-shapelet $\mathbf{f}$, a matrix $Dist$ that stores the multivariate distances between the shapelet and all $M$ time series in the dataset, and $Perc$ that determines the percentage of dimensions used to compute Equation 6. The algorithm sorts the matrix $Dist$ for each dimension separately (row by row)(line 2). Then the multivariate candidate threshold is computed (lines 4 and 5) as the mid-point between two successive distances for each dimension. Then, the condition in line 7 is modified to comply with Equation 6. The rest of the algorithm is unchanged.

## 4 Genes used in our experiments

The list of the genes used in our experiments for both viral infection and drug response datasets is provided in Table S.1.

**Algorithm S.3:** ComputeThreshold - Multivariate Information Gain

**Input**: A shapelet **f**; Dist: Matrix $N \times M$ where $N$ is the dimensions and $M$ is the number of time series; $Perc$
**Output**: A distance threshold $\Delta$

```
Initialize (tp,fp,tn,fn, maxIG)
SortedDist = Sort(Dist){Sort each dimension individually}
for j ← 1 to M − 1 do
    for k ← 1 to N do
        CandThr (k) = (SortedDist(k,j)+SortedDist(k,j+1)) / 2
    for i ← 1 to M do
        if Dist (i) ≤_Perc CandThr then {distance between f and T_i}
            if Class(T_i)==Class(f) then
                tp =tp +1
            else
                fp =fp +1
        else
            if Class(T_i)==Class(f) then
                tn =tn +1
            else
                fn =fn +1
    ELeft = −(tp/(tp+fp)) log(tp/(tp+fp)) − (tp/(tp+fp)) log(tp/(tp+fp))
    ERight = −(tn/(tn+fn)) log(tn/(tn+fn)) − (tn/(tn+fn)) log(tn/(tn+fn))
    InforGain = Entropy- ((tp+fp)/N) ELeft- ((tn+fn)/N) ERight
    if InforGain >maxIG then
        maxIG = InforGain
        Δ=CandThr
```

## 5 Evaluation of MSD method on the viral infection and drug response datasets using all genes

The MDS method was evaluated on the viral infection and drug repose datasets. The accuracy and the list of parameters used is provided in Table S.2.

## 6 Evaluation of MSD method on the viral infection and drug response datasets using a subset of genes

The MDS method was evaluated on the viral infection and drug repose datasets using a subset of genes. For the viral infection dataset, the subset has been chosen as the top genes from the ranked list (provided from the literature) that gives the highest accuracy. For the drug response datasets, the subset has been chosen by enumerating all combinations of genes and selecting a subset that gives the highest accuracy. For computational reasons, we could not enumerate all combinations of genes used in the Baranzini12 and Costa17 datasets.

Table S.1: The list of the genes used in our experiments for the viral infection and drug response datasets

| Dataset | Genes |
|---|---|
| H3N2 | RSAD2, IFI44L, SIGLEC1, LAMP3, IFIT1, IFI44, SERPING1<br>IFI27, ISG15, HERC5, LOC26010, IFI6, IFIT3, OAS3, OASL<br>XAF1, OAS1, LY6E, MS4A4A, TNFAIP6, CCL2, MX1, RTP4 |
| HRV | RSAD2, LAMP3, IFI44L, IFIT1, SIGLEC1, FI44, OAS3, SERPING1,<br>HERC5, ISG15, IFI6, INDO, MX1, IFIT3, OASL, LOC26010, CXCL10,<br>ATF3, OAS1, DDX58, LY6E, OAS2, CCL2, XAF1, IFIT2, SOCS1 |
| Baranzini3A | Caspase 2, Caspase 10, FLIP |
| Baranzini3B | Caspase 2, Caspase 3 , IRF4 |
| Baranzini6 | Caspase 7, Caspase 10, IRF2, IRF4, IRF6, IL-4Ra |
| Baranzini12 | Caspase 2, Caspase 3, Caspase 7, Caspase 10<br>Flip, IRF2, IRF4, IRF6, IL-4Ra, IL12Rb1, STAT4, MAP3K1 |
| Lin9 | Caspase 2, Caspase 3, Caspase 10, IL-4Ra<br>IL12Rb2, MAP3K1, IRF8, Jak2, RAIDD |
| Costa17 | Caspase 2, Caspase 3, Caspase 10, Caspase 5<br>MAP3K1, STAT4, IRF2, IRF4, IRF5, IRF8, BAX, Tyk2<br>IL-4Ra, IL-2Rg, IFN-gRb, IFNaR2, Jak2 |

Table S.2: MSD-based results on the viral infection and drug response datasets using all genes

| Dataset | Accuracy | Relative Accuracy | Coverage | Earliness | Perc | Pruning |
|---|---|---|---|---|---|---|
| H3N2 | 77.78 | 85.71 | 100 | 62.50 | 0.2 | Cover |
| HRV | 70.00 | 71.43 | 100 | 40.00 | 0.6 | Top10 |
| Baranzini3A | 70.00 | 73.91 | 95.83 | 46.26 | 0.7 | Top15 |
| Baranzini3B | 66.67 | 68.00 | 100 | 44.81 | 0.7 | Top15 |
| Baranzini6 | 70.83 | 70.83 | 100 | 42.86 | 0.6 | Top15 |
| Baranzini12 | 66.67 | 66.67 | 100 | 42.86 | 0.7 | Top10 |
| Lin9 | 67.86 | 69.57 | 100 | 44.00 | 0.9 | Top20 |
| Costa17 | 68.00 | 69.23 | 100 | 45.24 | 0.3 | Top5 |

Table S.3: MSD-based results on the drug response dataset using subset of genes that gives the highest accuracy

| Dataset | Number of genes | Accuracy | Relative Accuracy | Coverage | Earliness | Perc | Pruning |
|---|---|---|---|---|---|---|---|
| H3N2 | 11 | 80.00 | 87.50 | 88.89 | 64.29 | 0.1 | Cover |
| HRV | 1 | 71.43 | 75.00 | 100 | 38.89 | 0.1 - 1 | Top10 |
| Baranzini3A | 1 | 75.00 | 76.00 | 100 | 45.45 | 0.1 - 1 | Top10 |
| Baranzini3B | 2 | 75.00 | 76.19 | 100 | 44.05 | 0.6 - 1 | Top20 |
| Baranzini6 | 2 | 75.00 | 76.00 | 100 | 43.45 | 0.1 - 0.5 | Top10 |
| Lin9 | 3 | 81.82 | 82.61 | 100 | 43.43 | 0.7 | Top20 |

The accuracy and the list of parameters used is provided in Table S.3. For some cases, like Baranzini3A, the number of genes that gives the highest accuracy is one gene. In that case, the percentage of variables used to satisfy Equation 6 has no effect so that we report the percentage in the table as 0.1-1. Also when using 2 genes, like in Baranzini3B and Lin9, all percentages less than or equal to 0.5 have the same effect while all percentages greater than 0.5 have the same effect.

## 7 Comparison of distance threshold methods

The MDS method was evaluated on the viral infection and drug repose datasets using two different distance threshold methods. Namely, we compared the proposed distance threshold method information gain with the Chebyshev's inequality method. We note that using Chebyshev's inequality gives better relative accuracy, but on the other hand it gives worse coverage, which reduces the overall accuracy. We applied a paired t-test of the null hypothesis that the difference between the 1000 bootstrap accuracies for both methods are a random sample from a normal distribution with mean 0 and unknown variance, against the alternative that the mean is not 0. We applied the t-test at the 99% significance level. The results are shown in Table S.4. Using Chebyshev's inequality as a threshold method outperformed the information gain in only two datasets (Lin9 and Costa17) where in Costa17 the difference is not significant.

Table S.4: Comparison of distance threshold methods on the viral infection and drug response datasets

| Dataset | Information Gain | | | | Chebyshev's inequality | | | | $P$-value |
|---|---|---|---|---|---|---|---|---|---|
| | Acc | Rel Acc | Cov | Earliness | Acc | Rel Acc | Cov | Earliness | |
| H3N2 | 77.78 | 85.71 | 100 | 62.50 | 66.67 | 85.71 | 87.50 | 58.33 | 6.3e-062 |
| HRV | 70.00 | 71.43 | 100 | 40.00 | 55.56 | 100 | 57.14 | 60 | 4.9e-063 |
| Baranzini3A | 70.00 | 73.91 | 95.83 | 46.26 | 65.38 | 82.61 | 80.95 | 55.10 | 2.7e-052 |
| Baranzini3B | 66.67 | 68.00 | 100 | 44.81 | 62.96 | 76.92 | 84.81 | 53.90 | 1.4e-030 |
| Baranzini6 | 70.83 | 70.83 | 100 | 42.86 | 65.38 | 70.83 | 100.00 | 47.62 | 4.9e-052 |
| Baranzini12 | 66.67 | 66.67 | 100 | 42.86 | 66.67 | 69.23 | 100.00 | 45.58 | 0.036 |
| Lin9 | 67.86 | 69.57 | 100 | 44.00 | 68.18 | 77.27 | 91.67 | 50.55 | 3.2e-005 |
| Costa17 | 68.00 | 69.23 | 100 | 45.24 | 70.00 | 77.27 | 95.45 | 48.98 | 0.017 |

## 8 Comparison of utility score methods

The MSD method was evaluated on the viral infection and drug repose datasets using two utility score methods. Namely, we compared the proposed weighted information gain method with the weighted $F_1$ method. We applied a t-test to measure the significance of the difference at the 99% significance level. The results are shown in Table S.5. Using weighted $F_1$ score as a utility score, the method outperformed the weighted information gain in only one dataset (Baranzini6).

Table S.5: Comparison of utility score methods on the viral infection and drug response datasets

| Dataset | Weighted Information Gain | | | | Weighted $F_1$ score | | | | $P$-value |
|---|---|---|---|---|---|---|---|---|---|
| | Acc | Rel Acc | Cov | Earliness | Acc | Rel Acc | Cov | Earliness | |
| H3N2 | 77.78 | 85.71 | 100 | 62.50 | 68.26 | 71.43 | 100 | 42.71 | 7.2e-104 |
| HRV | 70.00 | 71.43 | 100 | 40.00 | 66.67 | 70.00 | 100 | 47.89 | 4.7e-163 |
| Baranzini3A | 70.00 | 73.91 | 95.83 | 46.26 | 66.67 | 68.00 | 100 | 43.94 | 3.9e-085 |
| Baranzini3B | 66.67 | 68.00 | 100 | 44.81 | 65.22 | 65.38 | 100 | 43.51 | 3.8e-071 |
| Baranzini6 | 70.83 | 70.83 | 100 | 42.86 | 72.00 | 72.00 | 100 | 42.86 | 9.6e-145 |
| Baranzini12 | 66.67 | 66.67 | 100 | 42.86 | 65.22 | 68.00 | 100 | 44.72 | 1.6e-093 |
| Lin9 | 67.86 | 69.57 | 100 | 44.00 | 65.38 | 66.67 | 100 | 43.51 | 0 |
| Costa17 | 68.00 | 69.23 | 100 | 45.24 | 67.00 | 68.00 | 100 | 43.51 | 1.6e-037 |
